# Supplementary material for: Intellectual disability‐associated gene ftsj1 is responsible for 2′‐O‐methylation of specific tRNAs
Source: EMBO Rep. 2020 Jun 18;21(8):e50095. doi: 10.15252/embr.202050095 (PMC7403668; doi:10.15252/embr.202050095)
Supplement: Supplementary file 1 — Appendix [file EMBR-21-e50095-s001.pdf]

Appendix

Contents on the first page

Appendix Figure S1. IP assays of FTSJ1-Flag. The empty vector was transfected as a control.

Appendix Figure S2. The knockout efficiency of *ftsj1* and *wdr6*.

Appendix Figure S3. Secondary structures of *S. cerevisiae* and *S. pombe* $tRNA^{Phe}$(GAA).

Appendix Table S1. List of potential FTSJ1-interacting proteins identified by IP-MS analysis.

Appendix Table S2. List of potential FTSJ1-interacting proteins identified by unnatural amino acid photocrosslinking-IP-MS analysis.

Appendix Table S3. Information about the 17 human cytoplasmic G37-tRNA species.

Appendix Table S4. Quantification of the Nm/A ratios in 16 G37-tRNAs after incubation with FTSJ1-WDR6 by UPLC-MS/MS analysis.

Appendix Table S5. Constructs used in this study.

Appendix Table S6. The biotinylated DNA probes for fishing tRNAs.

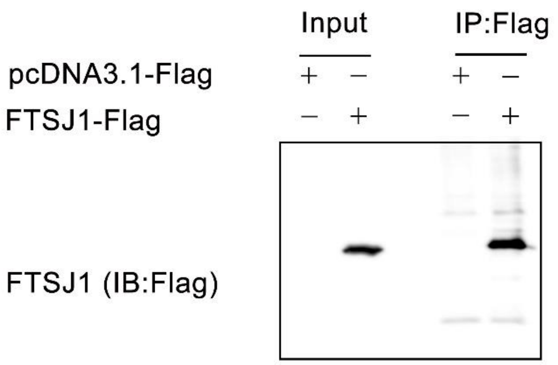

Appendix Figure S1. IP assays of FTSJ1-Flag. The empty vector was transfected as a control.

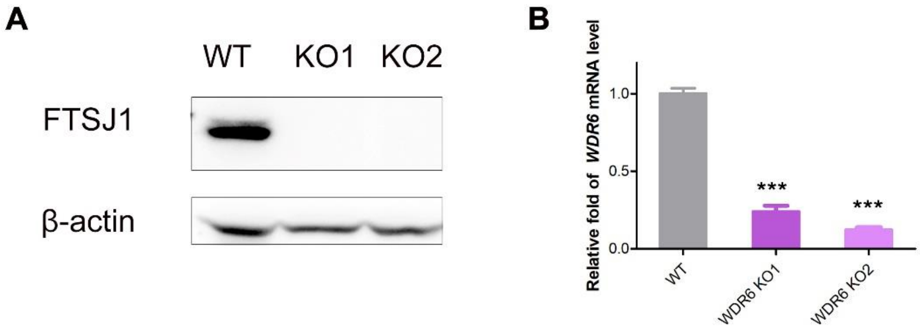

Appendix Figure S2. The knockout efficiency of *ftsj1* and *wdr6*.

(A) The western blotting to show *ftsj1* knockout efficiency. (B) QRT-PCR to show *wdr6* knockout efficiency. Error bars represent the standard deviation of three independent experiments. p values were determined using two-tailed Student's t-test for paired samples. $*p < 0.05$, $**p < 0.01$, $***p < 0.001$. n.s., no significance.

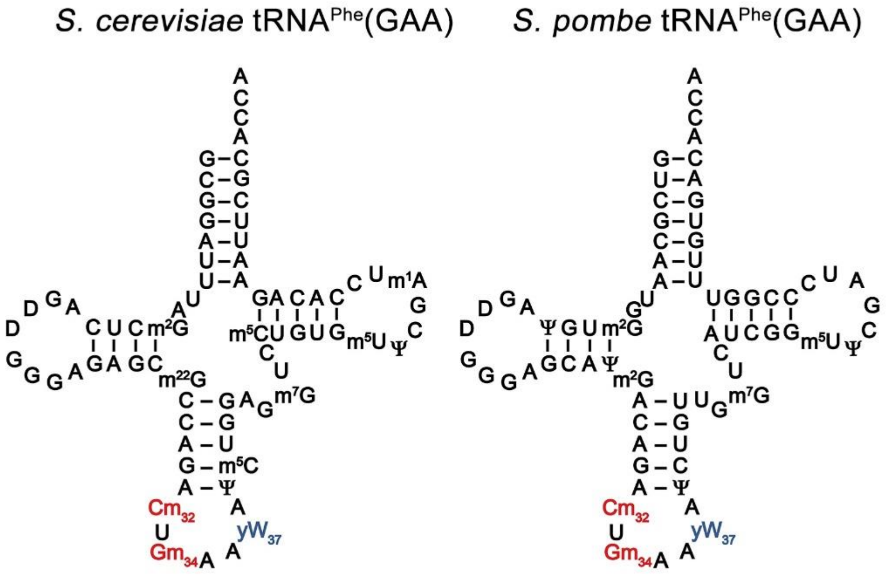

Appendix Figure S3. Secondary structures of *S. cerevisiae* and *S. pombe* tRNA^{Phe}(GAA).

Appendix Table S1. List of potential FTSJ1-interacting proteins identified by IP-MS analysis.

	Swiss-Prot ID	Gene Symbol	Description
1	P0DMV9	HSP1A	Heat shock 70 kDa protein 1B
2	B4DNT8		cDNA FLJ54370, highly similar to Heat shock 70 kDa protein 1
3	Q00610	CLTC	Clathrin heavy chain 1
4	P23246	SFPQ	Splicing factor, proline- and glutamine-rich
5	B4DI39		cDNA FLJ54328, highly similar to Heat shock 70 kDa protein 1
6	Q86VG2		Splicing factor proline/glutamine-rich (Polypyrimidine tract binding protein associated)
7	G8JLB6	HNRNPH1	Heterogeneous nuclear ribonucleoprotein H
8	Q15233	NONO	Non-POU domain-containing octamer-binding protein
9	V9HW22	HEL-S-72p	Epididymis luminal protein 33
10	P49750	YLPM1	YLP motif-containing protein 1
11	P78371	CCT2	T-complex protein 1 subunit beta containing TCP1, subunit 2 (Beta), isoform CRA_b
12	Q9UET6	FTSJ1	Putative tRNA (cytidine(32)/guanosine(34)-2'-O)-methyltransferase
13	Q8NF45	FLJ00353	FLJ00353 protein
14	P38646	HSPA9	Stress-70 protein, mitochondrial
15	O00571	DDX3X	ATP-dependent RNA helicase DDX3X
16	V9HW37	HEL-S-69	Epididymis secretory protein Li 69
17	P50990	CCT8	T-complex protein 1 subunit theta
18	P40227	CCT6A	T-complex protein 1 subunit zeta
19	P11021	HSPA5	Endoplasmic reticulum chaperone BiP
20	P22626	HNRNPA2B1	Heterogeneous nuclear ribonucleoproteins A2/B1
21	P35658	NUP214	Nuclear pore complex protein Nup214
22	Q5JP53	TUBB	Tubulin beta chain
23	Q53HV2		T-complex protein 1 subunit eta
24	P17987	TCP1	T-complex protein 1 subunit alpha
25	P49368	CCT3	T-complex protein 1 subunit gamma
26	F8VU51	YLPM1	YLP motif-containing protein 1
27	P50991	CCT4	T-complex protein 1 subunit delta
28	P68371	TUBB4B	Tubulin beta-4B chain
29	Q53FA3	HSPA1L	Heat shock 70 kDa protein 1-like (Fragment)
30	B7Z9L0		T-complex protein 1 subunit delta
31	Q9BVA1	TUBB2B	Tubulin beta-2B chain
32	P55795	HNRNPH2	Heterogeneous nuclear ribonucleoprotein H2
33	H0Y9K7	SFPQ	Splicing factor, proline- and glutamine-rich (Fragment)

34	Q92499	DDX1	ATP-dependent RNA helicase DDX1
35	O15042	U2SURP	U2 snRNP-associated SURP motif-containing protein
36	Q92841	DDX17	Probable ATP-dependent RNA helicase DDX17
37	Q7Z6Z7	HUWE1	E3 ubiquitin-protein ligase HUWE1
38	Q9Y3I0	RTCB	tRNA-splicing ligase RtcB homolog
39	J3KTA4	DDX5	Probable ATP-dependent RNA helicase DDX5
40	F8VPD4	CAD	CAD protein
41	P09651	HNRPA1	Heterogeneous nuclear ribonucleoprotein A1
42	Q02809	PLOD1	Procollagen-lysine,2-oxoglutarate 5-dioxygenase 1
43	P09651	HNRNPA1	Heterogeneous nuclear ribonucleoprotein A1
44	P04350;	TUBB4A	Tubulin beta-4A chain
45	O75533;	SF3B1	Splicing factor 3B subunit 1
46	P68363	TUBA1B	Tubulin alpha-1B chain 1A
47	Q9BQE3	TUBA1C	Tubulin alpha-1C chain
48	P14866	HNRNPL	Heterogeneous nuclear ribonucleoprotein L
49	Q15393;	SF3B3	Splicing factor 3B subunit 3
50	H0YBG7	HNRNPH1	Heterogeneous nuclear ribonucleoprotein H (Fragment)
51	Q01844;	EWSR1	RNA-binding protein EWS
52	P33993;	MCM7	DNA replication licensing factor MCM7
53	P61978	HNRNPK	Heterogeneous nuclear ribonucleoprotein K
54	Q92734	TFG	Protein TFG
55	Q9BTE3;	MCMBP	Mini-chromosome maintenance complex-binding protein
56	P33992	MCM5	DNA replication licensing factor MCM5
57	Q13509	TUBB3	Tubulin beta-3 chain
58	Q13435	SF3B2	Splicing factor 3B subunit 2
59	Q2NKY5	TUBB6	TUBB6 protein
60	Q8IXB1;	DNAJC10	DnaJ homolog subfamily C member 10
61	Q9H0D6	XRN2	5'-3' exoribonuclease 2
62	P52597	HNRNPF	Heterogeneous nuclear ribonucleoprotein F
63	P15924;	DSP	Desmoplakin
64	B2R802		cDNA, FLJ93681, highly similar to Homo sapiens small nuclear ribonucleoprotein polypeptide A (SNRPA), mRNA
65	A0A024QZD5	SNRP70	Small nuclear ribonucleoprotein 70kDa polypeptide (RNP antigen), isoform CRA_b
66	P04637	TP53	Cellular tumor antigen p53
67	Q4VCS5;	AMOT	Angiomotin
68	Q9C0J8;	WDR33	pre-mRNA 3' end processing protein WDR33
69	Q8WXF1	PSPC1	Paraspeckle component 1
70	P35637	FUS	RNA-binding protein FUS

71	P24928	POLR2A	DNA-directed RNA polymerase II subunit RPB1
72	B3KUD7	MCM7	NA replication licensing factor MCM7
73	Q9NNW5	WDR6	WD repeat-containing protein 6
74	P19338	NCL	Nucleolin
75	Q10570	CPSF1	Cleavage and polyadenylation specificity factor subunit 1
76	B4E3A4		cDNA FLJ57283, highly similar to Actin, cytoplasmic 2
77	Q86X55;	CARM1	Histone-arginine methyltransferase CARM1
78	Q99567;	NUP88	Nuclear pore complex protein Nup88
79	Q5RLJ0		CLE
80	P11940	PABPC1	Polyadenylate-binding protein 1
81	D3DWL9	CPSF1	Cleavage and polyadenylation specific factor 1, 160kDa, isoform CRA_a
82	P05387	RPLP2	60S acidic ribosomal protein P2
83	P63104	YWHAZ	14-3-3 protein zeta/delta
84	B4DZW4		Polyadenylate-binding protein
85	O14654;	IRS4	Insulin receptor substrate 4
86	P17066;	HSPA6	Heat shock 70 kDa protein 6
87	A0A024RDP4	PSPC1	Paraspeckle component 1, isoform CRA_b
88	P30876	POLR2B	DNA-directed RNA polymerase II subunit RPB2
89	Q4LE79	DSP	DSP variant protein (Fragment)
90	Q86YZ3;	HRNR	Hornerin
91	P49411;	TUFM	Elongation factor Tu, mitochondrial
92	Q8NCA5;	FAM98A	Protein FAM98A
93	P52272	HNRNPM	Heterogeneous nuclear ribonucleoprotein M
94	Q8IWX8	CHERP	Calcium homeostasis endoplasmic reticulum protein
95	P25705	ATP5A1	ATP synthase subunit alpha, mitochondrial
96	Q9BUF5	TUBB6	Tubulin beta-6 chain
97	Q99700;	ATXN2	Ataxin-2
98	P21333	FLNA	Filamin-A
99	O43143	DHX15	Pre-mRNA-splicing factor ATP-dependent RNA helicase DHX15
100	Q08211;	DHX9	ATP-dependent RNA helicase A
101	Q15149;	PLEC	Plectin
102	Q96I25	RBM17	Splicing factor 45
103	O43823;	AKAP8	A-kinase anchor protein 8
104	Q14444;	CAPRIN1	Caprin-1
105	Q00839	HNRNPU	Heterogeneous nuclear ribonucleoprotein U
106	Q52LJ0;	FAM98B	Protein FAM98B
107	P51991;	HNRNPA3	Heterogeneous nuclear ribonucleoprotein A3

Appendix Table S2. List of potential FTSJ1-interacting proteins identified by unnatural amino acid photocrosslinking-IP-MS analysis.

	Swiss-Prot ID	Gene Symbol	Description
1	Q9NNW5	WDR6	WD repeat-containing protein 6
2	O14654	IRS4	Insulin receptor substrate 4
3	P51532	SMARCA4	Transcription activator BRG1
4	Q9UET6	FTSJ1	Putative tRNA (cytidine(32)/guanosine(34)-2'-O)-methyltransferase
5	Q99729-3	HNRNPAB	Isoform 3 of Heterogeneous nuclear ribonucleoprotein A/B
6	O14979-2	HNRNPDL	Isoform 2 of Heterogeneous nuclear ribonucleoprotein D-like
7	P16403	HIST1H1C	Histone H1.2
8	P31483	TIA1	Nucleolysin TIA-1 isoform p40
9	Q01085-2	TIAL1	Isoform 2 of Nucleolysin
10	Q04837	SSBP1	Single-stranded DNA-binding protein, mitochondrial
11	Q00325-2	SLC25A3	Isoform B of Phosphate carrier protein, mitochondrial
12	Q9BTE3	MCMBP	Mini-chromosome maintenance complex-binding protein
13	P04350	TUBB4A	Tubulin beta-4A chain
14	Q9BUF5	TUBB6	Tubulin beta-6 chain
15	Q15366-2	PCBP2	Isoform 2 of Poly(rC)-binding protein 2
16	Q13247	SRSF6	Serine/arginine-rich splicing factor 6
17	P01605	IGKV1D-33	Immunoglobulin kappa variable 1D-33
18	Q01650	SLC7A5	Large neutral amino acids transporter small subunit 1
19	Q7Z2W4	ZC3HAV1	Zinc finger CCCH-type antiviral protein 1
20	P10809	HSPD1	60 kDa heat shock protein, mitochondrial
21	P07900-2	HSP90AA1	Isoform 2 of Heat shock protein HSP 90-alpha OS
22	Q71U36	TUBA1A	Tubulin alpha-1A chain
23	P12236	SLC25A6	ADP/ATP translocase 3
24	Q9BQ39	DDX50	ATP-dependent RNA helicase DDX50
25	Q13243	SRSF5	Serine/arginine-rich splicing factor 5
26	O00264	PGRMC1	Membrane-associated progesterone receptor component 1
27	P20674	COX5A	Cytochrome c oxidase subunit 5A, mitochondrial
28	P63173	RPL38	60S ribosomal protein L38
29	P30954	OR10J1	Olfactory receptor 10J1
30	O60783	MRPS14	28S ribosomal protein S14, mitochondrial
31	Q9GZQ3	COMMD5	COMM domain-containing protein 5
32	P51843	NR0B1	Nuclear receptor subfamily 0 group B member 1
33	Q9NX58	LYAR	Cell growth-regulating nucleolar protein

34	P82932	MRPS6	28S ribosomal protein S6, mitochondrial
35	O75908-3	SOAT2	Isoform 3 of Sterol O-acyltransferase 2
36	Q9UNA1	ARHGAP26	Rho GTPase-activating protein 26
37	Q8N5I4	DHRSX	Dehydrogenase/reductase SDR family member on chromosome X
38	Q99832	CCT7	T-complex protein 1 subunit eta
39	Q9UKF6	CPSF3	Cleavage and polyadenylation specificity factor subunit 3
40	P02745	C1QA	Complement C1q subcomponent subunit A
41	Q14246	ADGRE1	Adhesion G protein-coupled receptor E1
42	P52292	KPNA2	Importin subunit alpha-1
43	Q8WUZ0-2	BCL7C	Isoform 2 of B-cell CLL/lymphoma 7 protein family member C
44	P40227	CCT6A	T-complex protein 1 subunit zeta
45	P35613	BSG	Basigin
46	Q6PK04	CCDC137	Coiled-coil domain-containing protein 137
47	Q9BU76	MMTAG2	Multiple myeloma tumor-associated protein 2
48	P10243	MYBL1	Myb-related protein A
49	Q9Y5S1	TRPV2	Transient receptor potential cation channel subfamily V member 2
50	Q08999	RBL2	Retinoblastoma-like protein 2
51	O94979-8	SEC31A	Isoform 8 of Protein transport protein
52	Q9Y383	LUC7L2	Putative RNA-binding protein Luc7-like 2
53	P13639	EEF2	Elongation factor 2
54	P33993	MCM7	DNA replication licensing factor MCM7
55	Q9Y4P3	TBL2	Transducin beta-like protein 2
56	P61619	SEC61A1	Protein transport protein Sec61 subunit alpha isoform 1
57	Q68CZ2	TNS3	Tensin-3
58	O14980	XPO1	Exportin-1
59	Q9Y2X0	MED16	Mediator of RNA polymerase II transcription subunit 16
60	Q6P158	DHX57	Putative ATP-dependent RNA helicase DHX57
61	O14917	PCDH17	Protocadherin-17
62	P25705	ATP5A1	ATP synthase subunit alpha, mitochondrial
63	Q5T9A4	ATAD3B	ATPase family AAA domain-containing protein 3B
64	Q9Y2I1	NISCH	Nischarin
65	P57678	GEMIN4	Gem-associated protein 4

Appendix Table S3. Information about the 17 human cytoplasmic G37-tRNA species.

17 G37-tRNAs		Site 32	Site 34
	$tRNA^{Arg}$(ACG)	C32	A34
$tRNA^{Arg}$	-(CCG)	U32	C34
	-(UCG)	C32	U34

tRNA^{Cys}(GCA)	tRNA^{Cys}(GCA)	C32	G34
tRNA^{His}(GUG)	tRNA^{His}(GUG)	U32	G34
tRNA^{Leu}	tRNA^{Leu}(AAG)	U32	A34
	-(CAA)	C32	C34
	-(CAG)	U32	C34
	-(UAA)	C32	U34
	-(UAG)	U32	U34
tRNA^{Phe}(GAA)	tRNA^{Phe}(GAA)	C32	G34
tRNA^{Pro}	tRNA^{Pro}(AGG)	U32	A34
	-(CGG)	U32	C34
	-(UGG)	U32	U34
tRNA^{Trp}(CCA)	tRNA^{Trp}(CCA)	C32	C34
tRNA^{Tyr}	tRNA^{Tyr}(AUA)	C32	A34
	-(GUA)	C32	G34

Appendix Table S4. Quantification of the Nm/A ratios in 16 G37-tRNAs after incubation with FTSJ1-WDR6 by UPLC-MS/MS analysis.

tRNAs	Am/A%	Cm/A%	Gm/A%	Um/A%	m^1G/A%
m^1G37_tRNA^{Arg}(ACG)	n.d.				5.21±0.24
m^1G37_tRNA^{Arg}(CCG)		n.d.			0.34±0.01
m^1G37_tRNA^{Arg}(UCG)				n.d.	0.92±0.08
m^1G37_tRNA^{Cys}(GCA)			n.d.		1.38±0.06
m^1G37_tRNA^{His}(GUG)			n.d.		6.67±0.15
m^1G37_tRNA^{Leu}(AAG)	n.d.				6.89±0.67
m^1G37_tRNA^{Leu}(UAG)				n.d.	7.05±0.28
m^1G37_tRNA^{Leu}(CAG)		n.d.			3.01±0.45
m^1G37_tRNA^{Leu}(UAA)				n.d.	4.99±0.28
m^1G37_tRNA^{Pro}(AGG)	n.d.				8.53±0.32
m^1G37_tRNA^{Pro}(CGG)		n.d.			8.37±0.22
m^1G37_tRNA^{Pro}(UGG)				n.d.	2.70±0.10
m^1G37_tRNA^{Phe}(GAA)			0.75±0.04		2.26±0.20
m^1G37_tRNA^{Trp}(CCA)		n.d.			5.73±0.11
m^1G37_tRNA^{Tyr}(GUA)			n.d.		5.47±0.50
m^1G37_tRNA^{Tyr}(AUA)	n.d.				3.35±0.12

The results are the average of three independent repeats with standard deviations indicated. n.d., not detected.

Appendix Table S5. Constructs used in this study.

Proteins	Tag	Position	Vector
**For expression in insect cells**			
FTSJ1	His$_6$	C-terminus	pFastbac1
WDR6	GST	N-terminus	pFastbac1

TrmD	His_6	N-terminus	pET28a
**For expression in HEK293T cells**			
FTSJ1	Flag	C-terminus	pcDNA3.1
FTSJ1-△N19, -△C39, -220, -241, -256 and -Asn221DIZPK			
WDR6	HA	C-terminus	pcDNA3.1
**For immunofluorescence analysis**			
FTSJ1	Flag	C-terminus	pcDNA3.1

Appendix Table S6. The biotinylated DNA probes for fishing tRNAs.

**tRNAs**	**Probes**
$tRNA^{Arg}$(ACG)	5′biotin-CAGACGCGTTATCCATTGCGCCACTGGCCC
$tRNA^{Arg}$(CCG)	5′biotin-TGGCGACCACGAAGGGACTCGAACCCTCAA
$tRNA^{Arg}$(UCG)	5′biotin-TGGCGAGCCAGCCAGGAGTCGAACCTGGAA
$tRNA^{Cys}$(GCA)	5′biotin-TCAAATGCTCTACCACTGAGCTATGCCCCC
$tRNA^{His}$(GUG)	5′biotin-GCAGAGTACTAACCACTATACGATCACGGC
$tRNA^{Leu}$(UAA)	5′biotin-CCAACGCCTTAACCACTCGGCCATCCCGGT
$tRNA^{Leu}$(AAG) and -(UAG)	5′biotin-CAGCGCCTTAGACCGCTCGGCCACGCTACC
$tRNA^{Leu}$(CAG)	5′biotin-CAGCGCCTTAGACCGCTCGGCCATCCTGAC
$tRNA^{Pro}$(AGG), -(CGG) and -(UGG)	5′biotin-GCGAGAATCATACCCCTAGACCAACGAGCC
$tRNA^{Trp}$(CCA)	5′biotin-TCAGACGCGCTACCATTGCGCCACGAGGTC
$tRNA^{Phe}$(GAA)	5′biotin-CTAACGCTCTCCCAACTGAGCTATTTCGGC
$tRNA^{Tyr}$(AUA)	5′biotin-CCTCTGCTCTACCAGCTGAACTATTGAAGG
$tRNA^{Tyr}$(GUA)	5′biotin-CCTCCGCTCTACCAACTGAGCTATCGAAGG
elongator $tRNA^{Met}$(CAU)	5′biotin-CTGACGCGCTACCTACTGCGCTAACGAGGC
$tRNA^{Ala}$(AGC)	5′biotin-GCACGCGCTCTACCACTGAGCTATACCCCC
$tRNA^{Gly}$(GCC)	5′biotin-GCGAGAATTCTACCACTGAACCACCCATGC
$tRNA^{Gly}$(CCC)	5′biotin-TCTTGCATGATACCACTACACCAGCGGCGC
$tRNA^{Gln}$(UUG)	5′biotin-CCAGAGTGCTAACCATTACACCATGGGGCC
