# Supplementary material for: Intellectual disability‐associated gene ftsj1 is responsible for 2′‐O‐methylation of specific tRNAs
Source: EMBO Rep. 2020 Jun 18;21(8):e50095. doi: 10.15252/embr.202050095 (PMC7403668; doi:10.15252/embr.202050095)
Supplement: Supplementary file 2 — Expanded View Figures PDF [file EMBR-21-e50095-s002.pdf]

Expanded View Figures

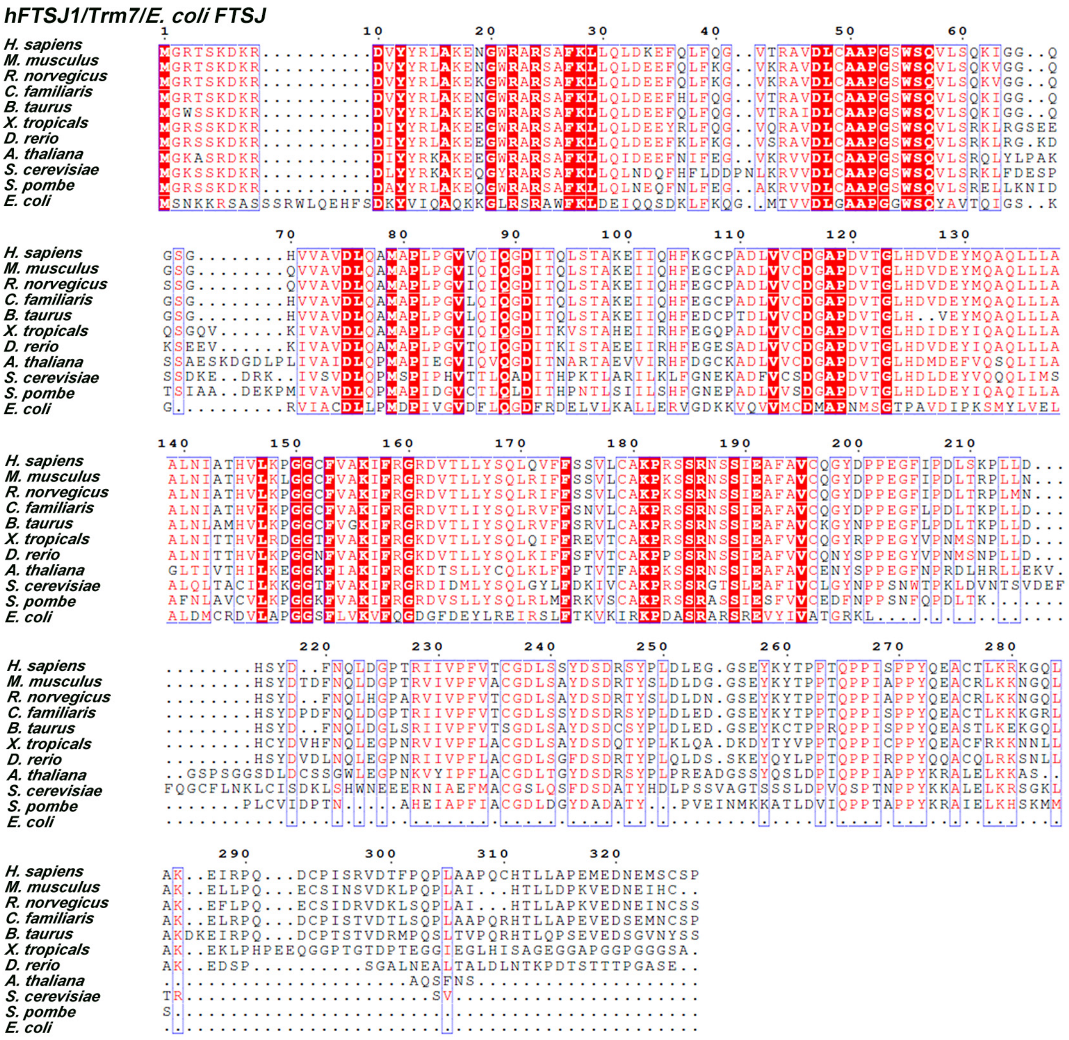

Figure EV1. Sequence alignments of human FTSJ1, eukaryotic Trm7, and *E. coli* FTSJ/RrmJ.

H. sapiens, Homo sapiens; M. musculus, Mus musculus; R. norvegicus, Rattus norvegicus; C. familiaris, Canis familiaris; B. taurus, Bos taurus; X. tropicalis, Xenopus tropicalis; D. rerio, Danio rerio; A. thaliana, Arabidopsis thaliana; S. cerevisiae, Saccharomyces cerevisiae; S. pombe, Schizosaccharomyces pombe; E. coli, Escherichia coli.

Source data are available online for this figure.

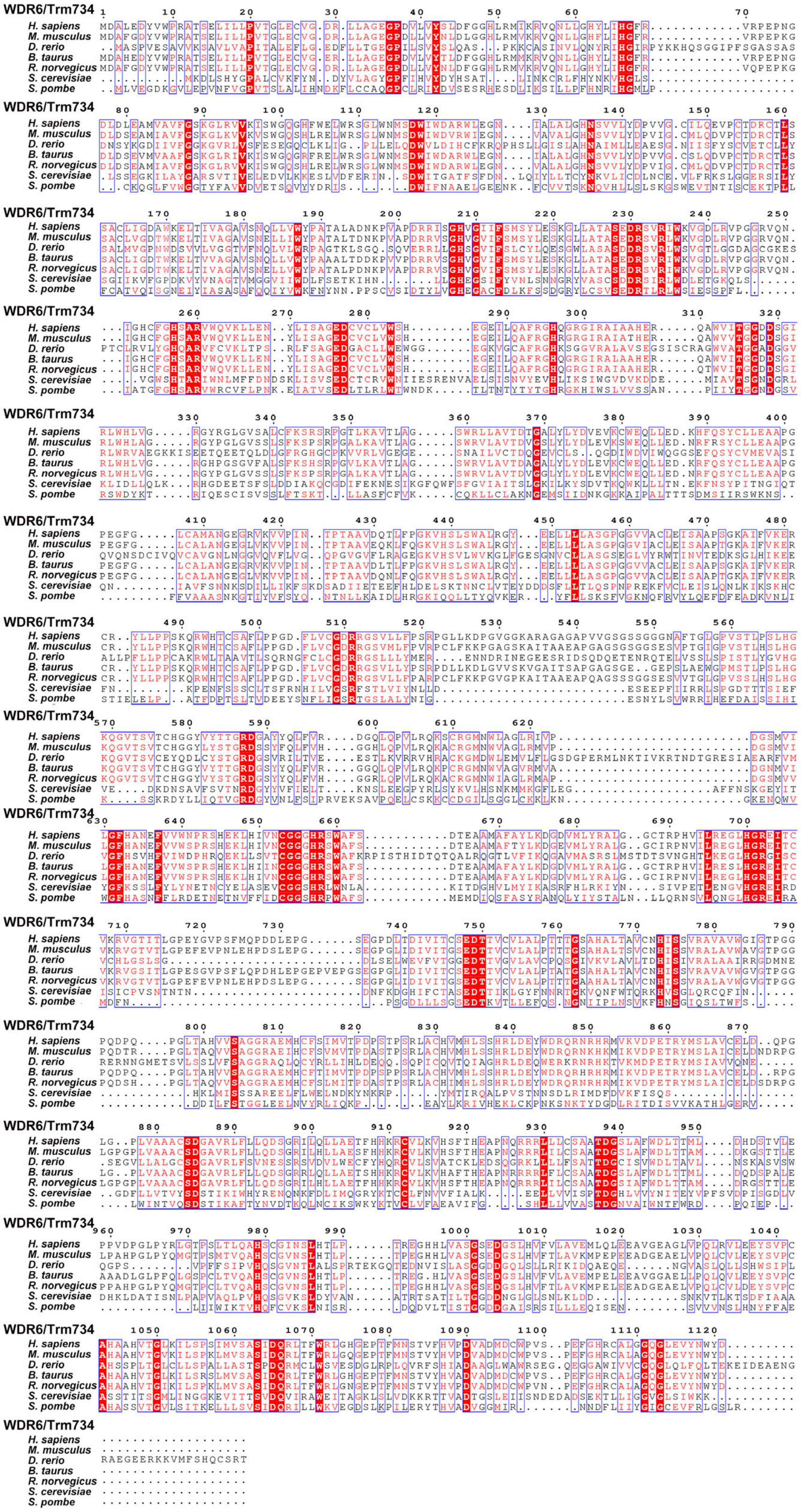

Figure EV2. Sequence alignments of WDR6 or Trm734 from different species.

S. cerevisiae and *S. pombe* contain Trm734, the auxiliary protein for Trm7. Some higher eukaryotes, such as *H. sapiens*, *M. musculus*, *D. rerio*, *B. taurus*, and *R. norvegicus*, contain the Trm734 homolog, WDR6.

Source data are available online for this figure.

 © The Authors

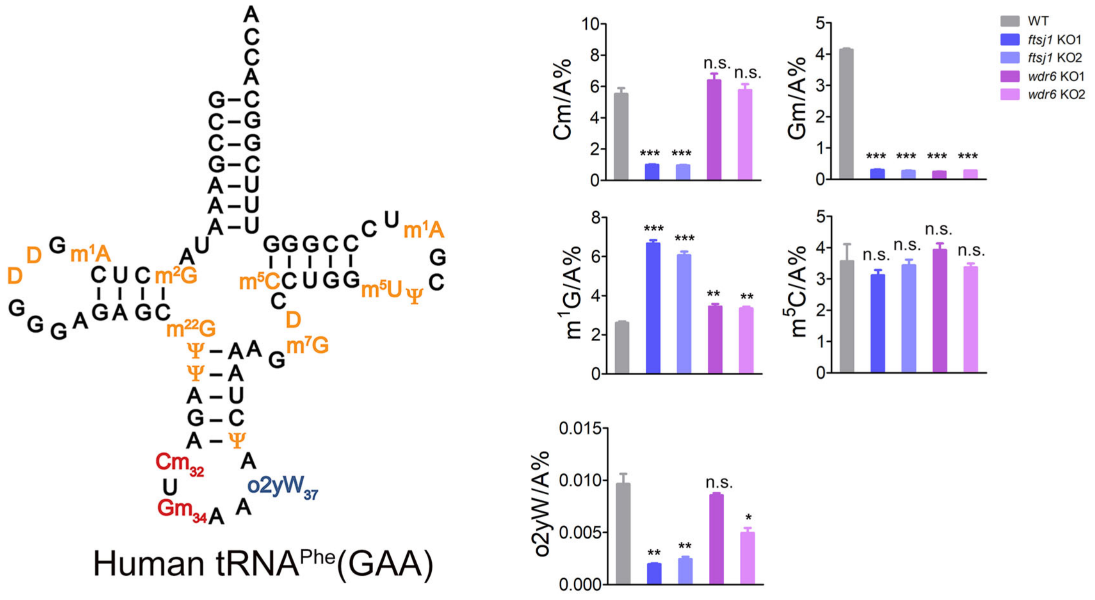

Figure EV3. Knockout of *ftsj1* and/or *wdr6* affects Cm32, Gm34, m^{1}G37, and o2yW37 levels of tRNA^{Phe}(GAA).

Quantification of the Cm/A, Gm/A, m^{1}G/A, m^{5}C/A, and o2yW/A ratios in endogenous tRNA^{Phe}(GAA) isolated from WT, *ftsj1* KO, and *wdr6* KO cell lines by UPLC-MS/MS analysis. Error bars represent the standard deviation of three independent experiments. *P* values were determined using two-tailed Student's *t*-test for paired samples. $*P < 0.05$, $**P < 0.01$, $***P < 0.001$. n.s., no significance.

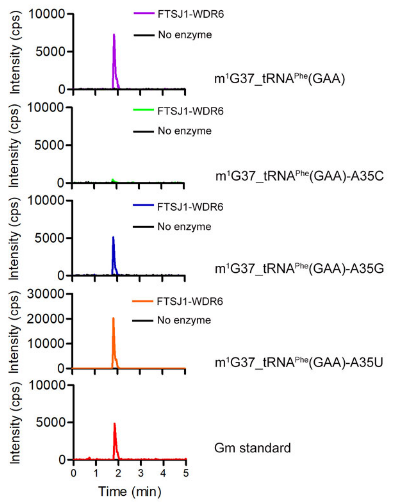

Figure EV4. UPLC-MS/MS analysis of Gm34 of m^{1}G37_tRNA^{Phe}(GAA), m^{1}G37_tRNA^{Phe}(GAA)-A35C, m^{1}G37_tRNA^{Phe}(GAA)-A35G and m^{1}G37_tRNA^{Phe}(GAA)-A35U after incubation with or without FTSJ1-WDR6.

2 μl of Gm standard (1 ng/ml) was loaded as control. cps, counts per second.

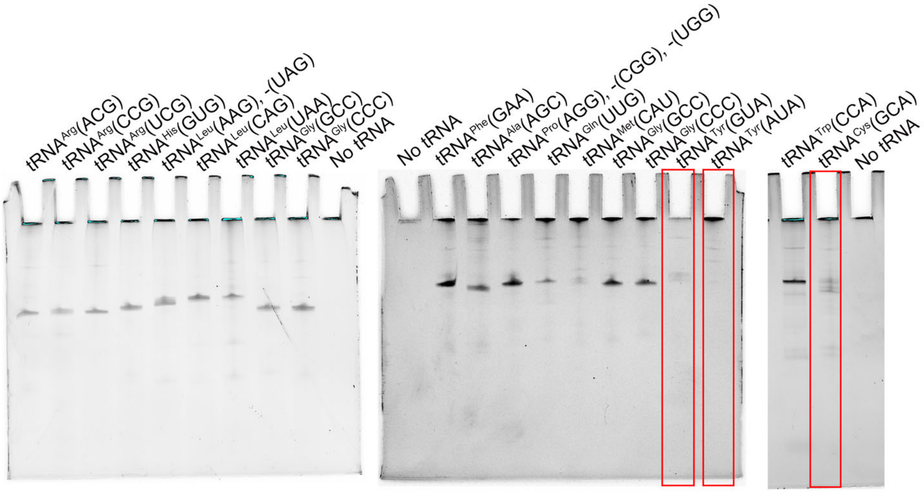

Figure EV5. Analysis of the purity of the fished tRNAs by biotinylated DNA probes using urea PAGE.

Among them, tRNA^{Cys}(GCA), tRNA^{Tyr}(GUA), or tRNA^{Tyr}(AUA) could not be purified (in red box).

 © The Authors
